# Gastritis cystica profunda recurrence after surgical resection: 2-year follow-up

**DOI:** 10.1186/1477-7819-12-133

**Published:** 2014-04-30

**Authors:** Lei Wang, Hua Yan, Da-Chun Cao, Li Huo, Hai-Zhong Huo, Bing Wang, Ying Chen, Hai-Lin Liu

**Affiliations:** 1 Department of Gastroenterology, the Ninth People’s Hospital Affiliated to the School of Medicine, Shanghai Jiaotong University, Shanghai 200011, China; 2 Department of Surgery, the Ninth People’s Hospital Affiliated to the School of Medicine, Shanghai Jiaotong University, Shanghai 200011, China; 3 Department of Pathology, the Ninth People’s Hospital Affiliated to the School of Medicine, Shanghai Jiaotong University, Shanghai 200011, China

**Keywords:** Gastritis cystica profunda, Gastric cancer, Endoscopic ultrasonography

## Abstract

**Background:**

Gastritis cystica profunda (GCP) is an uncommon disease characterized by multiple cystic gastric glands within the submucosa of the stomach.

**Case description:**

Here, we present a case of a 63-year-old man with intermittent epigastric discomfort in whom gastroscopy revealed multiple irregular elevated nodular lesions with smooth surfaces at the anterior of the antrum. Surgical resection of the nodular lesions was performed, and the diagnosis of gastritis cystica profunda (GCP) was confirmed by histological examination. Another elevated nodular lesion approximately 10 mm in diameter with an ulcer was found on the gastric side of the remnant stomach near the resection side from 6 to 24 months after the surgical resection. Endoscopic ultrasonography (EUS) and repeated biopsies of the new elevated lesion were performed. Homogeneous, anechoic masses originating from the submucosa without gastric adenocarcinoma in histological examination showed GCP recurrence may occur.

**Conclusions:**

We report a case of GCP recurrence within 6 months after surgical resection. GCP should be considered in the differential diagnosis of elevated lesions in the stomach.

## Background

Gastritis cystica profunda (GCP) is an uncommon disease characterized by multiple cystic gastric glands within the submucosa of the stomach. GCP seemed to be limited to previously operated stomachs in prior reports, but it has been found in unoperated stomachs in several recent articles [1-3]. Here, we report a case of GCP that occurred in a patient who had not previously undergone gastric surgery, and the GCP recurred in just 6 months after surgical resection.

## Case presentation

**Figure 1:**
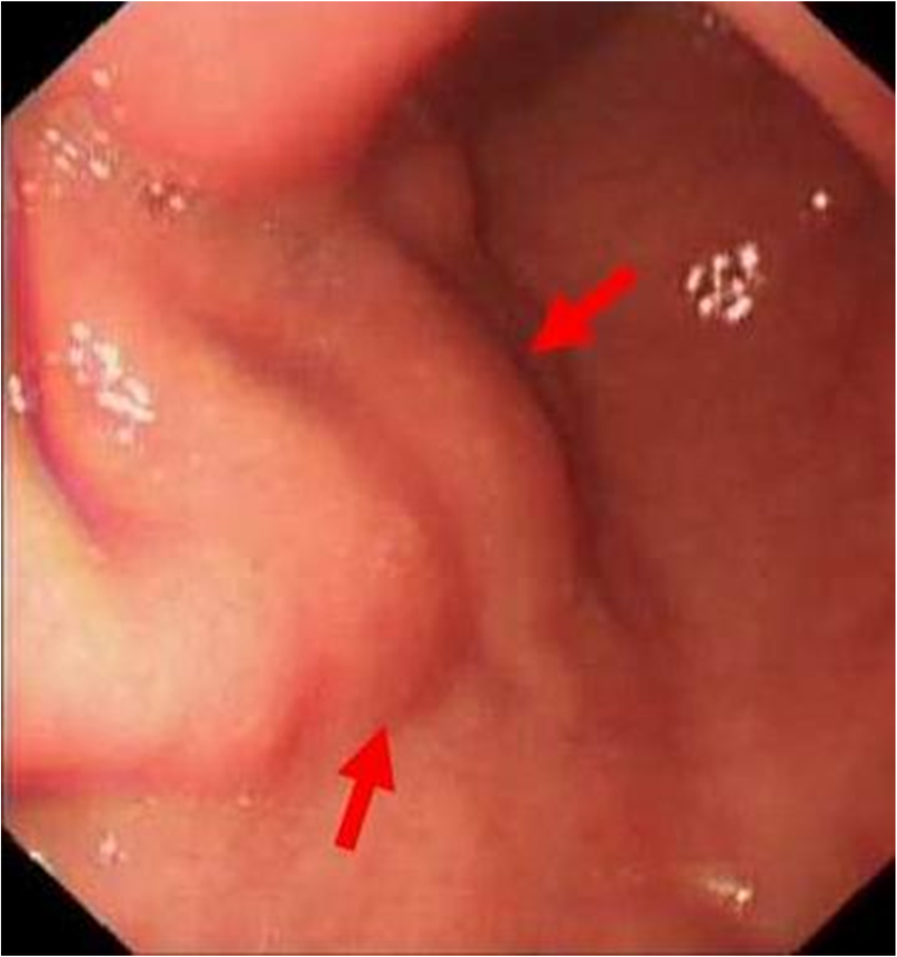
Gastroscopy revealing multiple irregular elevated nodular lesions (arrows) with smooth surfaces in the anterior portion of the antrum.

**Figure 2:**
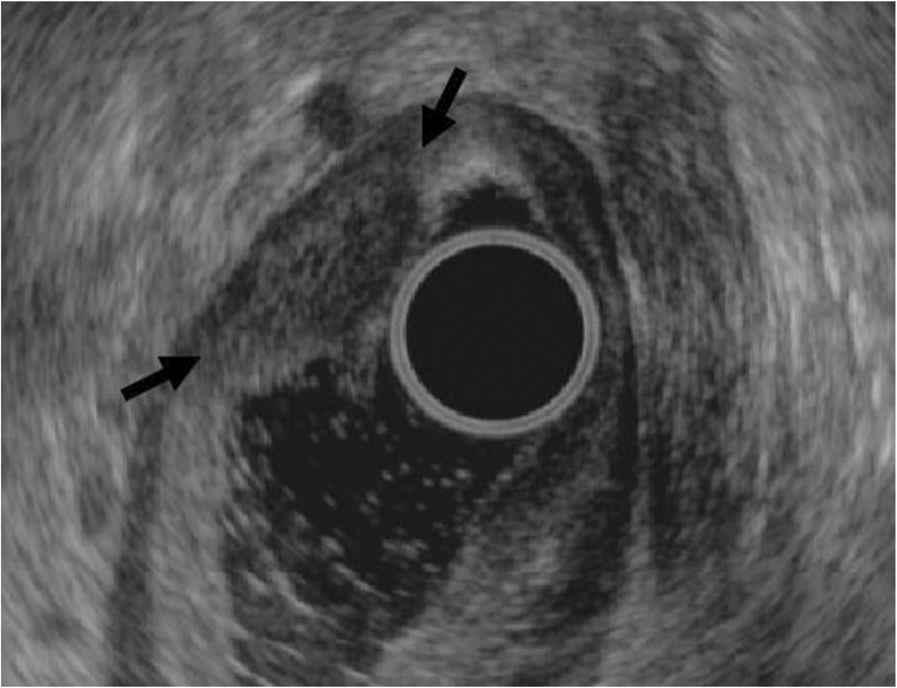
Endoscopic ultrasonography showing a hypoechoic mass without clear margins (arrows) in the submucosa.

A 63-year-old man was admitted to the gastroenterology department for intermittent epigastric discomfort. He had no history of gastric operations. Blood tests and physical examination showed no abnormalities except the CEA level of serum was 7.68 ng/mL. Gastroscopy revealed multiple irregularly elevated nodular lesions with smooth surfaces on the anterior portion of the antrum (Figure 1). Two biopsies revealed normal mucosa. Endoscopic ultrasonography (EUS) showed a hypoechoic mass in the submucosa without clear margins (Figure 2). Abdominal computed tomography showed no significant gastric or perigastric lesions of the stomach.

**Figure 3:**
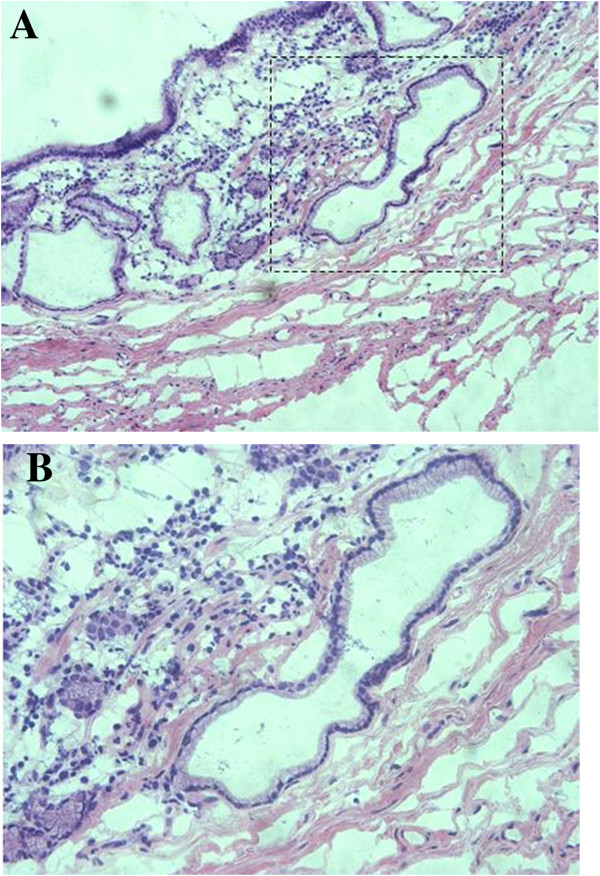
Histological examination of the nodular lesions showing dilated cystic glands in the muscularis mucosa and submucosa (hematoxylin and eosin staining; (A) × 100; (B) × 200).

Exploratory laparotomy was performed with the patient’s claiming because malignancy was suspected according to the abnormal CEA level. Two elevated nodular lesions approximately 10 mm and 15 mm in diameter were identified in the anterior portion of the antrum during the operation. The nodular lesions were surgically resected. A histological examination showed dilated cystic glands in the muscularis mucosa and submucosa (Figure 3), and a diagnosis of GCP was established.

**Figure 4:**
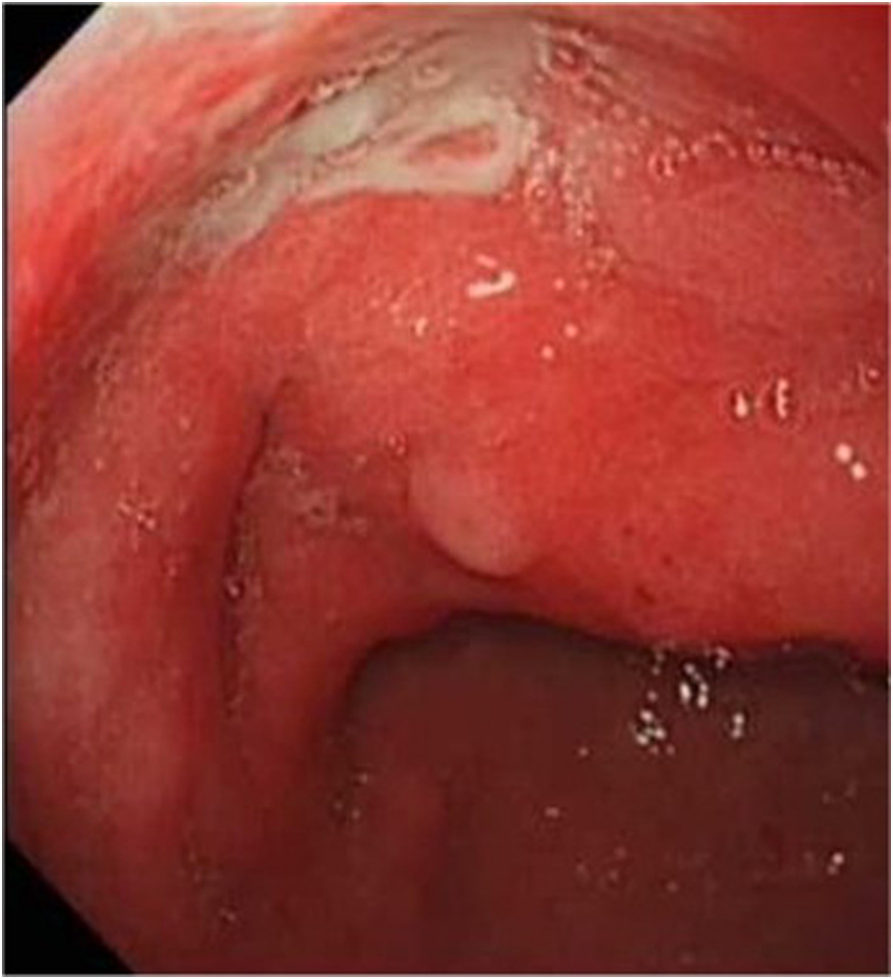
Follow-up gastroscopy at 6 months after the surgical resection finding another elevated nodular lesion with an ulcer on the gastric side of the remnant stomach.

**Figure 5:**
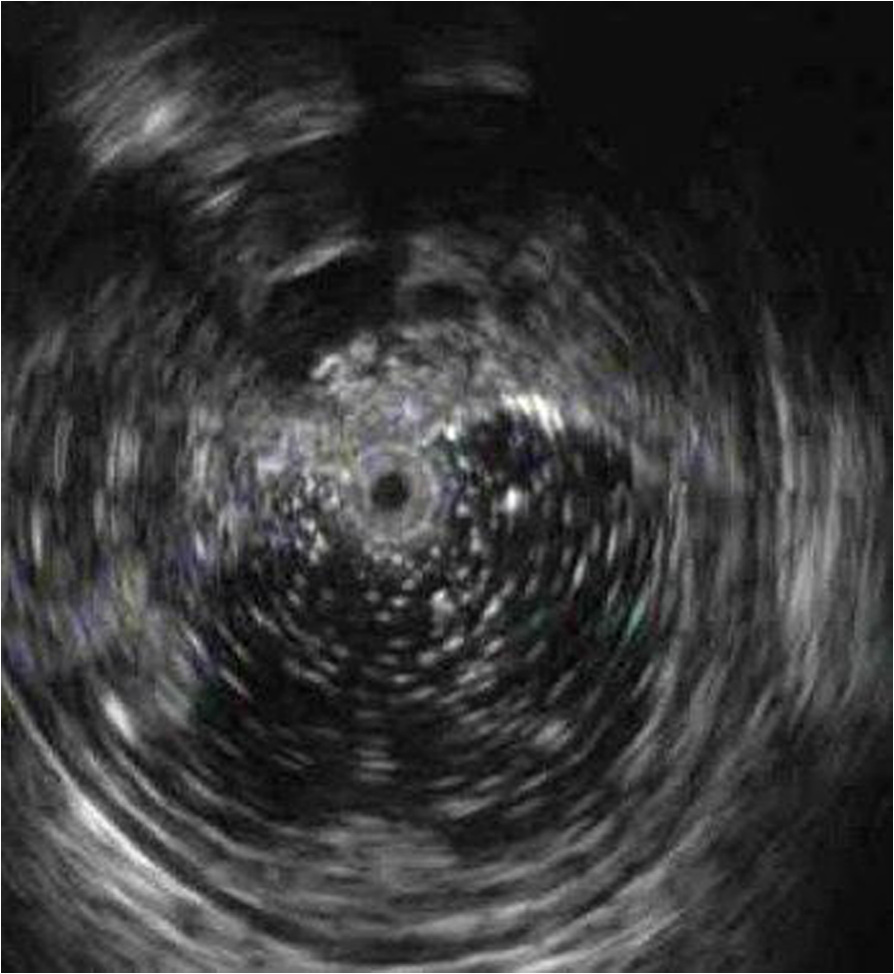
EUS of the new lesion showing several homogeneous, anechoic masses originating from the submucosa.

Follow-up gastroscopies were performed at 6 months, 12 months, and 24 months after the surgical resection, and another elevated nodular lesion approximately 10 mm in diameter with an ulcer was found on the gastric side of the remnant stomach near the resection side (Figure 4). Another EUS of the new lesion was performed showing several homogeneous, anechoic masses originating from the submucosa (Figure 5). Three repeated biopsies of the newly elevated lesion were performed, and histological examination confirmed chronic gastritis without gastric adenocarcinoma. So the GCP recurrence may occur after surgical resection according to the EUS.

## Discussion

GCP is a rare disease that was first described by Littler ER in 1972 [4] and is characterized by multiple cystic gastric glands within the submucosa of the stomach. The lesion usually occurs at gastroenterostomy sites; however, it has also been found in the stomachs of patients who have not previously undergone gastric surgery in several other articles [1-3]. GCP tends to occur more frequently in the presence of gastric cancers [2,3,5-8]; in a pathological study of 10,728 patients with gastric cancer, it was found in 161 patients [8]. There are some reports of GCP coexisting with Ménétrier disease (MD) [9,10] or gastric inverted hyperplastic polyps (IHP) [11]. To the best of our knowledge, this report is the first case report of GCP recurrence within a short time after surgical resection.

GCP may present as elevated lesions in gastroscopy and should be differentiated from early gastric cancers, submucosal tumors, giant gastric folds, or polyps. GCP can cause massive upper gastrointestinal hemorrhages in some cases [12,13], and it may be a cause of gastric outlet obstruction when accompanied by adenocarcinoma [3]. EUS revealed a hypoechoic mass without clear margins in the submucosa in the present case, which differs from homogeneous, hypoechoic, and multilocular polypoid masses in other cases [1-3]. The diagnosis of GCP should be confirmed by histological examination.

GCP is usually considered a benign lesion, but it can be a precancerous gastric lesion. Its malignant potential should be valued because GCP accompanied by gastric adenocarcinoma has been documented in several reports [2,3,5-8]. Ten-year follow-up of a case with GCP in an unoperated patient revealed low-grade dysplasia, and the sizes of both the GCP and the adenoma overlying it increased during the follow-up period [14].

The mechanism of GCP is not very clear. Roepke TK et al. [15] demonstrated that targeted deletion of the *Kcne2* gene in mice can cause a severe gastric preneoplastic phenotype comprising GCP. Kim L et al. [16] found that the Epstein-Barr virus (EBV) in situ hybridization revealed a positive reaction at the dysplastic area in a case of GCP associated with gastric carcinoma with lymphoid stroma. Choi MG et al. [8] found that the EBV-positive rate was significantly higher in a GCP gastric cancer group (31.1%) than in a non-GCP gastric cancer group (5.8%), which suggested that GCP was significantly associated with EBV-positive gastric cancers and that EBV infection may play a role in dysplastic changes associated with GCP. Mongolian gerbil model animals infected with *Helicobacter pylori* could develop GCP, gastric ulcers, and focal dysplasia. Additionally, the *H. pylori* cag-pathogenicity island-dependent immunological response may trigger GCP [17].

## Conclusions

In conclusion, although GCP is extremely rare, it should be considered in the differential diagnosis of elevated lesions in the stomach.

## Consent

Written informed consent was obtained from the patient for the publication of this report and any accompanying images.

## Abbreviations

EBV: Epstein-Barr virus; EMR: Endoscopic mucosal resection; EUS: Endoscopic ultrasonography; GCP: Gastritis cystica profunda; IHP: Inverted hyperplastic polyp; MD: Ménétrier disease.

## Competing interests

The authors declare that they have no competing interests.

## Authors’ contributions

WL wrote the article; WL, YH, CDC, and HL did the gastroscopy and endoscopic ultrasonography; HHZ and WaB did the operation; CY did the pathological tests; LHL made the final approval of the article. All authors read and approved the final manuscript.
